# Activated Carbon Produced by Pyrolysis of Waste Wood and Straw for Potential Wastewater Adsorption

**DOI:** 10.3390/ma13092047

**Published:** 2020-04-27

**Authors:** Katarzyna Januszewicz, Paweł Kazimierski, Maciej Klein, Dariusz Kardaś, Justyna Łuczak

**Affiliations:** 1 Department of Energy Conversion and Storage, Chemical Faculty, Gdańsk University of Technology, Narutowicza 11/12, 80–233 Gdańsk, Poland; 2 Institute of Fluid Flow Machinery, Polish Academy of Sciences, 80-233 Gdańsk, Poland; pawel.kazimierski@imp.gda.pl (P.K.); maciejklein@imp.gda.pl (M.K.); dariusz.kardas@imp.gda.pl (D.K.); 3 Energy Research Institute @ NTU (ERI@N), Nanyang Technological University, 50 Nanyang Drive, Singapore 637553, Singapore; 4 Department of Process Engineering and Chemical Technology, Chemical Faculty, Gdańsk University of Technology, Narutowicza 11/12, 80–233 Gdańsk, Poland; justyna.luczak@pg.edu.pl

**Keywords:** waste biomass, pyrolysis, activated carbon, physical and chemical activation, biochar

## Abstract

Pyrolysis of straw pellets and wood strips was performed in a fixed bed reactor. The chars, solid products of thermal degradation, were used as potential materials for activated carbon production. Chemical and physical activation processes were used to compare properties of the products. The chemical activation agent KOH was chosen and the physical activation was conducted with steam and carbon dioxide as oxidising gases. The effect of the activation process on the surface area, pore volume, structure and composition of the biochar was examined. The samples with the highest surface area (1349.6 and 1194.4 m^2^/g for straw and wood activated carbons, respectively) were obtained when the chemical activation with KOH solution was applied. The sample with the highest surface area was used as an adsorbent for model wastewater contamination removal.

## 1. Introduction

Biomass, as an effective source of energy, is one of the alternatives to fossil fuels, which can be utilised by direct burning or indirect thermal treatment. Thermal degradation processes, like pyrolysis or gasification, were designed to convert the organic matter into a variety of products including gases, oils and chars [1]. During pyrolysis, biomass is thermally decomposed in the oxygen-depleted conditions with temperatures between 300 and 1200 °C. This temperature range is seen as a main advantage when compared with gasification and combustion processes [2,3]. The temperature of the gasification processes is set in a range of 600–1200 °C (typically above 700 °C), whereas combustion of biomass is carried out up to 1300 °C [4,5]. In these conditions, biomass is decomposed into lighter chemical compounds due to several reactions, mostly of endothermic nature. As a consequence, volatile matter is generated providing liquid and gaseous products used mainly as fuels, however, by-products in the form of char are also obtained. The quality and quantity of each product are highly influenced by the temperature of the process, residence time, heating rate, and size and shape of the biomass particles [6].

Utilisation of waste biomass materials in pyrolysis process is a part of a current trend of closed cycle production to achieve more sustainable processes and products. The residue charred material formed during pyrolysis has much higher carbon content (% wt.) in comparison to the substrate, since moisture, volatiles, and most of the compounds with non-carbon heteroatoms are removed during the thermal treatment. Char with low commercial value formed from waste biomaterials can be activated to produce adsorbents for various industrial processes or used directly as a fuel [7]. The final surface properties and functionalities of the char are determined by the nature of pyrolyzed precursors and the type of activation process. In this regard, a variety of raw materials, as well as methods to enhance properties of the char, were investigated to produce effective and low-cost products with high sorption properties. Chars were produced from waste of wooden and non-wooden origin [8,9], including agricultural residues [10], rice husk, palm shells [11], coconut shells [11,12] , apple and cherry pulp, plum pulp and stones, and olive stones among others [13,14,15,16,17]. The high surface area and ability to influence the pore size and distribution during the production process makes adsorbents from waste biomass suitable for areas as different as the separation and purification of gases, water treatment, energy storage and catalysis, among others [18,19,20,21].

Activation processes increase porosity, enhance surface areas and modify chemical properties by introducing a variety of surface sites such as carboxylic, phenolic, hydroxylic and carbonyl groups [22]. The most important reason for the use of different technics of activation is to increase a partially blocked porous surface by using agents to remove the trapped products of incomplete pyrolysis. The activation can be either physical or chemical in nature. In the first attempt, the material is exposed to high temperatures (800–900 °C) and treated with steam or CO_2_ in a nitrogen or argon atmosphere. Thermal treatment is a basic method of improving the sorption parameters of the char, which also forms new functional groups on the surface [18]. Oxidation with agents mainly increases the amount of surface functional groups [23], but at the same time, accompanied by temperature, expands the porous structure of the material by removing trapped gases and oils (products of pyrolysis process) and oxidises existing functional groups. Steam, as an activation agent, increases the hydrophilicity and changes the properties of the char by taking part in the generation of additional oxidation agents like CO and CO_2_. Physical treatment with CO_2_ is often preferred over steam due to its low reactivity, which makes the process easier to control, thus obtaining more uniform porosity [24].

For the chemical activation of char, KOH, ZnCl_2,_ NaOH, H_3_PO_4_ and K_2_CO_3_ impregnation can be applied, accompanied by high temperatures. By using this method, the hydrophilic properties of biochar can be improved and the N/C and H/C (aromaticity) ratio increased [18]. Chemical activation with alkaline reagents, especially KOH, is widely used, providing materials with very high surface area and defined micropore size distribution [25]. This process is usually performed at high temperatures, e.g., 650–950 °C [26]. The activation mechanism can be described by the following reactions: dehydratation (2KOH = K_2_O + H_2_O); water–gas reaction (C + H_2_O = H_2_ + CO); water–gas shift reaction (CO + H_2_O = H_2_ + CO_2_); and carbonate formation (K_2_O + CO_2_ = K_2_CO_3_) [26]. Above 700 °C metallic potassium is formed due to reduction by carbon or hydrogen. The comparison of two typical alkaline activation agents, KOH and NaOH, revealed that in the similar condition of activating the process the biomass samples prepared with NaOH had lower surface area and micropore volumes than those with KOH [27]. However, the chemical activation process must be followed by an additional treatment to eliminate chemicals used [28]. 

Nevertheless, the main challenge in the production of activated carbon (AC) is development of the economically justified method to obtain products with given surface properties using low cost materials [29,30,31,32]. In this regard, the aim of this study is to compare different technics for increasing the surface area and porosity of the biochar obtained from waste beech wood and wheat and rye straw pellets. Both raw materials are by-products of agriculture and the wood industry. For example, in Poland, the overproduction of straw in relation to agricultural demand reaches about 10 million tons per year, which is equivalent to around 6 million tons of coal [33]. The additional valuable utilization method of waste straw could be pyrolysis and valorization through its activation. Additionally, the mode of action of the activated carbon with the highest surface area was determined as a potential adsorbent for the removal of model contaminant—Rhodamina B (RhB) from aqueous solutions. 

## 2. Materials and Methods 

### 2.1. Materials

**Figure 1:**
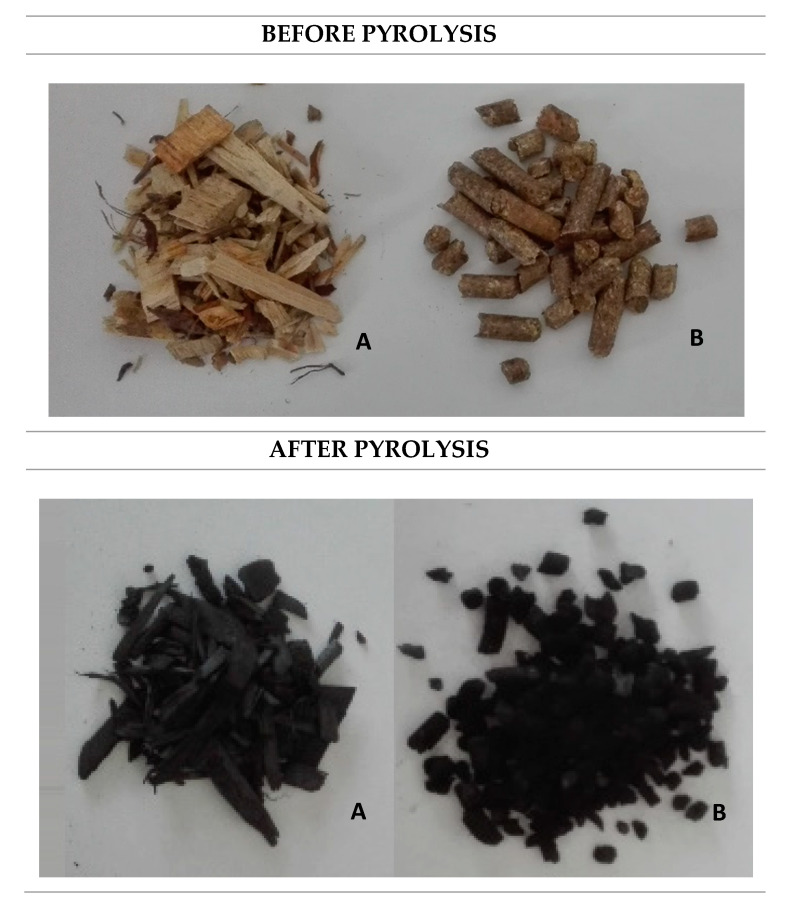
Raw materials before and after pyrolysis: (**A**) beech wood chips; and (**B**) straw pellets.

Two types of biomass from the Polish local market, wood and straw, were chosen as substrates for pyrolysis and biochar production. The pellet straw and wood chips were used as examples of agricultural waste. Typically, the straw pellets are a heterogeneous mixture of various waste straw, like wheat and rye. This pellet is commonly used in Polish agriculture and is commercially available, therefore such biomass was chosen for the experiments. The beech wood residues in the form of chips with a particle size distribution of about 3 × 2 × 2 cm and mixed wheat and rye straw pellets (commercial product used e.g., as animal bedding) with a length of 5–25 mm and a diameter of 6 mm were used (Figure 1).

### 2.2. Pyrolysis and Activation Process of Biochar

#### 2.2.1. Pyrolysis Process of Biomass

**Figure 2:**
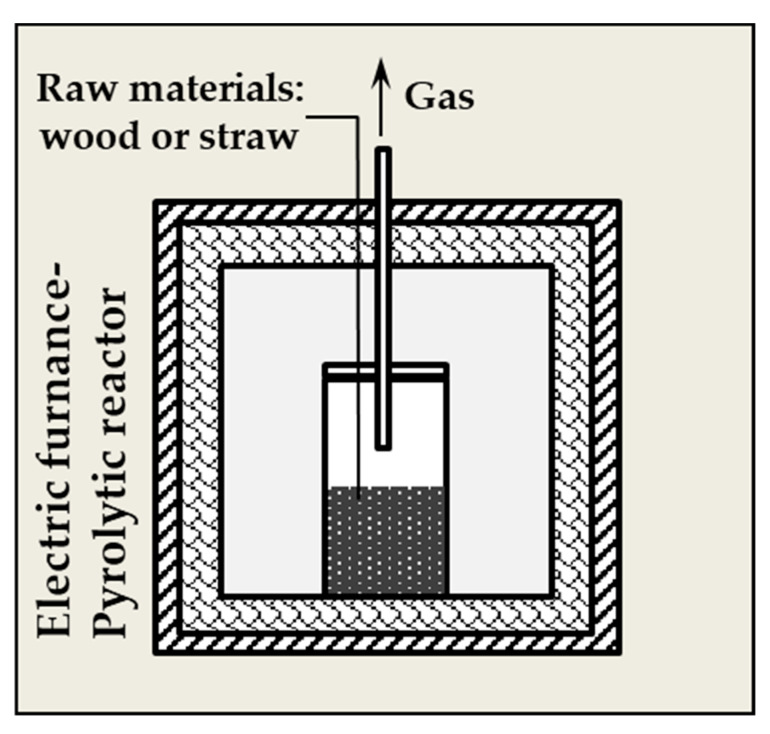
Scheme of the pyrolysis reactor.

The fast pyrolysis process of the biomass samples (about 100 g) was performed in a steel reactor (250 mL) placed in an electrical furnace (Figure 2) at 800 °C for 30 min; the heating rate was 100 °C/min. The process took place under limited access of air without inert gas. During the process, the gases and tars produced passed through 4 scrubbers filled with isopropanol. 

The amount of char used in the experiment was 23.8 g for straw pellets and 22.5 g for wood chips. The chars obtained in that way were ground and sieved to obtain a product with a particle size distribution of 1–2 mm, and then dried. The obtained biochar was subjected to the activation process controlled by gas flow and furnace temperature. Three different methods of activation were chosen: chemical using KOH, physical by steam and CO_2_ treatment. In each case, treatment of the biochar by the selected agent was performed under high temperatures (800–850 °C for about 1 h) according to the procedures described below. 

#### 2.2.2. Chemical Activation of the Biochar

The chemical activation with KOH was carried out in a laboratory-scale reactor. The sample (char) was mixed with a solid KOH with a mass ratio of 1:2 and heated at 850 °C. The KOH dosage was chosen based on the previous investigation [30], which revealed that an increase in the KOH: char mass ratio provides a higher surface area of the material. The activation process involved heating the chars for 1 h at 850 °C (heating rate of 10 °C/min) under an N_2_ atmosphere (flow rate of 50 mL/min). After the activation process, the product was cooled under N_2_, and then 250 mL of distilled water was added. The suspension was sonicated for 60 min and left for 24 h for AC sedimentation. After filtration, the resulting carbon was washed with water and 5% HCl until a neutral pH of the washed solution was achieved. The final product was dried at 80 °C overnight and ground well into a powder.

#### 2.2.3. Physical Activation of the Biochar

Both physical activations, performed using steam and CO_2_ as the activation agents, were carried out in the tubular furnace at 850 °C. The biochar samples were treated by CO_2_ or steam with the flow rate of 10 dm^3^/h for 0.5 or 1 h. The molar ratio of the biochar to the activating gas of 1:0.5 and 1:1 was applied. The amount of the biochar needed for these molar ratios determination was calculated based on the carbon content (in % wt.) in the material. For that purpose, the elemental analysis of the biochar samples was performed.

### 2.3. Characterisation of the Materials 

Elemental composition of the substrates and biochar was performed using the CHNS-O analyser Flash 2000 (Thermo Scientific, Waltham, MA, USA). For thermogravimetric (TG) analysis of the raw materials the SDT Q600 thermogravimeter (TA instruments, New Castle, DE, USA) was used. The mass loss of the samples as a function of temperature (25–500 °C) at 15 °C/min heating rate was applied. Moisture content was determined by using a moisture analyser (Mettler Toledo, Greifensee, Switzerland). The calorific value was analysed by the PN-ISO 1928:2002 method with using a KL-12 MN calorimeter (PRECYZJA-BIT, Bydgoszcz, Poland). The Brunauer–Emmett–Teller (BET) surface area and pore size distribution of the activated carbons was determined by N_2_ absorption–desorption isotherms at 77 K using a Micromeritics Gemini V200 Shimadzu (Kyoto, Japan) analyser. The morphology of the materials was studied by scanning electron microscopy (SEM) analysis, performed using a Hitachi SU3500, Tokyo, Japan. 

### 2.4. Adsorption Studies

A stock solution of Rhodamine B (1000 mg/dm^3^) prepared in deionised water was diluted to a concentration of 20–100 mg/dm^3^ to prepare a series of standard solutions for the calibration curve. In order to determine the rate of RhB adsorption, the experiments were conducted with different amounts of adsorbent at a constant particle size, pH, temperature and initial concentration of RhB. The activated carbon was added at different doses in the range from 2.7 to 20.6 mg into 20 mL of RhB solution (30 mg/dm^3^) [34]. The experiments were carried out at 25 °C (Pol-Eco incubator, Gdańsk, Poland). The suspensions were shaken thoroughly for 30 min using a mechanical shaker (IKA- WERKE, OS 10 B, Yellow line, Staufen im Breisgau, Germany) at 120 rpm. The samples were then filtered at the same time intervals and the concentrations of the dye solutions were analysed by UV-VIS spectrophotometer at 553 nm (UV-VIS Evolution 220; Thermo Scientific, Waltham, MA, USA). 

## 3. Results and Discussion

Before the pyrolysis process was performed in a fixed bed reactor, the composition and properties of the substrates were characterised by elemental and TG analyses. The characteristics of the straw pellets and waste wood are shown in Table 1. The wood samples contained 67.9 wt.% volatiles, 8.4 wt.% moisture and 2.3 wt.% of ash. Elemental analysis revealed that wood is composed of 45.0 wt.% carbon, 6.4 wt.% hydrogen, 1.3 wt.% nitrogen and 47.3 wt.% oxygen. For comparison, waste straw contained more volatiles (85.6 wt.%) and more ash 5.9 (wt.%), but less moisture (6.7 wt.%). According to the elemental analysis the content of carbon, hydrogen, nitrogen and oxygen in the straw was 49.0 wt.%, 6.9 wt.%, 1.7 wt.% and 36.6 wt.%, respectively. Carbon content data (in % wt.), taken from elemental analysis, were than used for calculation of the amount of CO_2_ and steam used for the physical activation experiments (gas to carbon molar ratio was chosen to be 1:0.5 and 1:1). 

**Figure 3:**
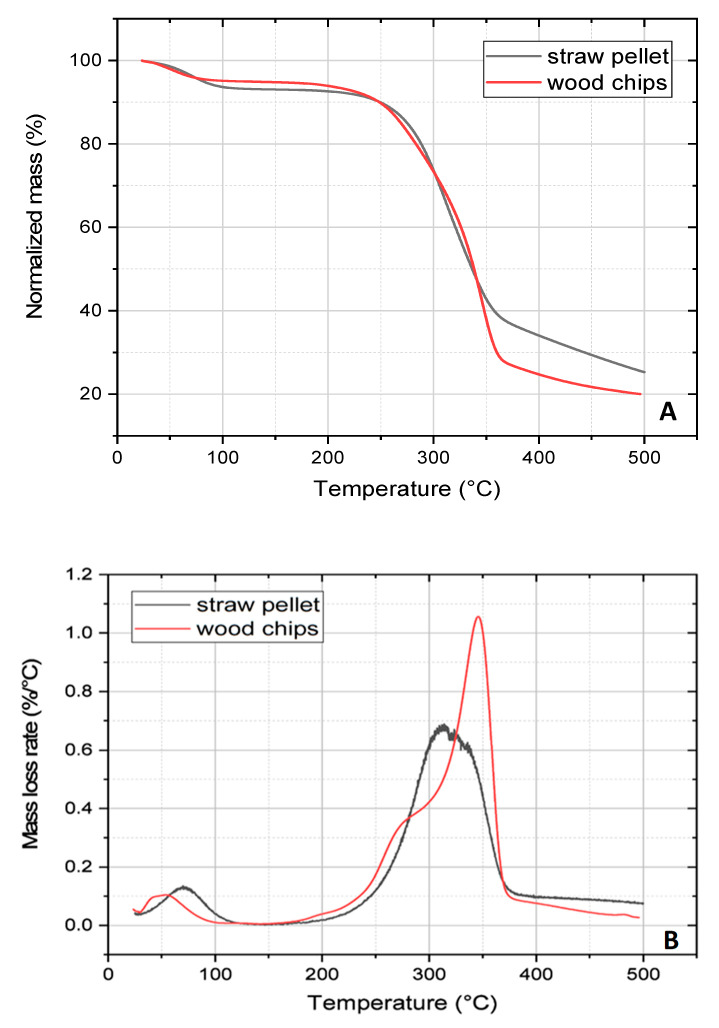
(**A**) Thermal analysis of the straw and wood samples; (**B**) derivative of mass loss in time for straw and wood samples obtained at 15 °C/min under a nitrogen atmosphere.

The TG analysis of the raw materials (Figure 3) provided initial characteristics of the pyrolysis process. In Figure 3A mass loss of both biomass samples as a function of temperature (from 25 to 500 °C) obtained for a heating rate of 15 °C/min) is presented. In the contrary to the abovementioned results (volatile matter) these measurements revealed lower mass loss in straw pellets than in wood samples. The differences in these results are related with distinct conditions of both measurements. Especially, temperature is a key parameter which for volatile matter determination was much higher (850 °C), providing higher mass loss. The shape and height of the peaks in the TG curves represent differences in the degradation intensity and substrate compositions. Thermal changes of the materials, presented in a graph, revealed three main steps of the process. These are water release, gas-forming reactions and cracking of vapours to form tars and gases (hydrocarbons). Small peaks below 100 °C were related to the evaporation of water from both samples. Comparing the properties of the samples, the decomposition of straw pellets occurs in the temperature range of 220–380 °C, similar to the wood sample. However, the maximum of the main peak representing the degradation of wood was detected at 350 °C and this peak was higher than that obtained for straw (maximum at 300 °C). The main difference in both characteristics is that the derivative of mass loss (DTG) curve (Figure 3B), representing pyrolysis of the wood, contains a main degradation peak as well as a smaller one appearing on the left side (wood degrades gradually). The straw pellet degradation, in turn, revealed only one main characteristic peak. This could be related to the higher uniformity of the straw composition (degradation temperature of hemicellulose is about 250–350 °C) [35]. The right side shoulder of the main peak, in turn, represents the cellulose degradation (usually taking place in the range from 325 to 400 °C) [35]. Lignin, as the third main component of the biomass, is also part of both biomass samples, influencing the conversion into volatiles. The DTG experiment revealed that most of the volatile matter in both raw materials is formed up to 380–400 °C. This information was taken into account when designing the pyrolysis experiments performed in the semi-technical scale. Nevertheless, these data cannot be crucial since a much higher temperature (above 700 °C) is needed to completely remove the volatile fraction from pores [26].

### 3.1. Characterisation of Carbon Materials

**Figure 4:**
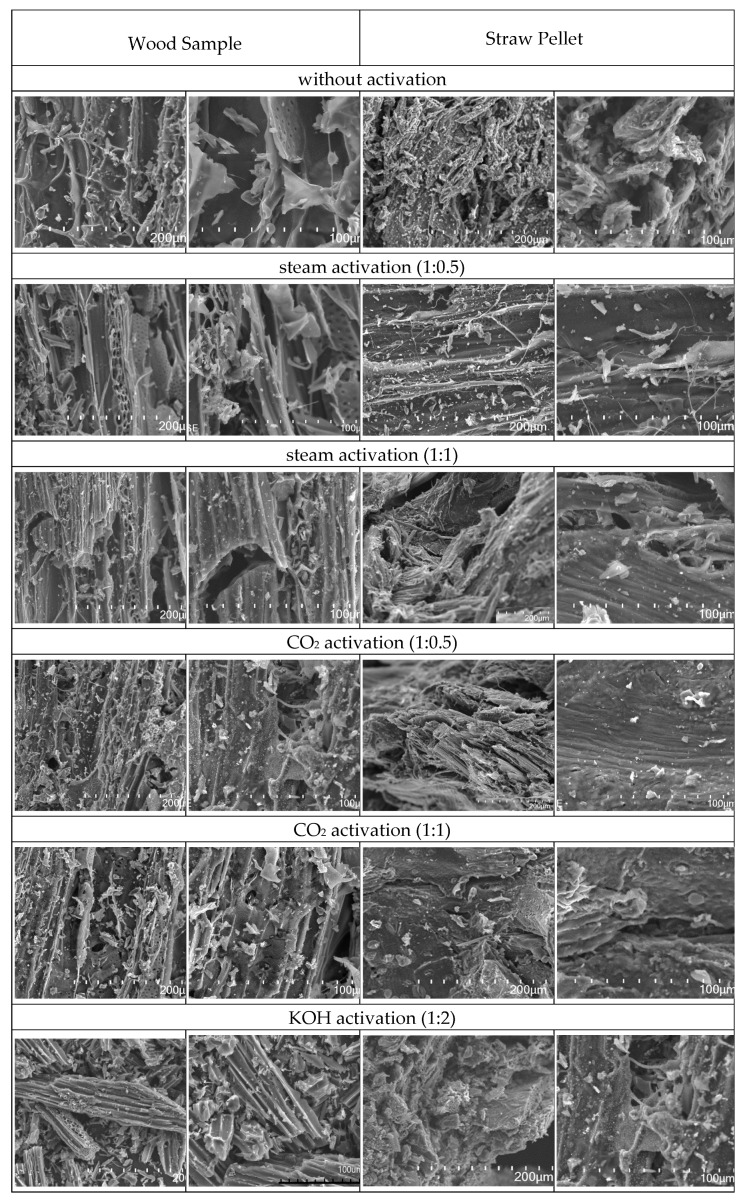
Scanning electron microscopy (SEM) micrographs presenting comparison of wood and straw samples after different activation methods. The molar ratio of biochar to the activating gas or mass ratio of biochar to KOH are shown in parentheses.

The biochar samples obtained as a result of the pyrolysis process, prepared in a fixed bed reactor at 800 °C, were characterised, taking into account chemical composition (Table 1) and surface area (Table 2). As expected, the elemental analysis confirmed an increase in the carbon content up to 76.2 wt.% (from 49 wt.% before pyrolysis) and 74.0 wt.% (from 45 wt.% before pyrolysis) in the straw and wood chars, respectively. As a consequence of the substrate thermal decomposition, the relative content of hydrogen decreased (released in the gas phase), whereas nitrogen increased (up to 3.4–3.5 wt.%). The surface area of these samples (reference samples shown in Table 2) was relatively low: 3.1 m^2^/g and 27.3 m^2^/g for straw and wood char, respectively. The obtained biochar was then subjected to the chemical and physical activation processes described above. The change in the surface area due to the application of different activation methods is also presented in Table 2. The difference in the surface area and pore volume arises from the type of activation agent, treatment method and raw material structure. Analysis of the results presented in Table 2 indicate that the surface area of the activated carbons prepared using the straw pellets is relatively higher. Differences in cell construction and a lack of fibrous structures in the straw pellet sample (as shown by SEM analysis presented in Figure 4) probably resulted in better adsorption parameters (higher pore volume and surface area). Wood is composed of relatively more densely packed cells, and is therefore a harder material (hardness above 500 KG/cm^2^). Nevertheless, the form of the raw material also matters: wood was used as strips, whereas straw was used in the form of pellets. The pellet structure enabled relatively higher penetration of the activation agents during chemical or physical treatment, thus also determining the final properties of the material. 

The results of the surface analysis revealed that the mechanism of chemical activation was more effective in comparison to the use of other activation agents. In this method, penetration of the material with KOH, and thus modification of the carbon surface, was achieved to a higher extent than in the case of physical activation. The sample with the highest surface area was obtained by the chemical activation of biochar obtained from straw pellets (1349.6 m^2^/g). In comparison, analogous samples prepared using wood strips revealed a surface area of 1194.4 m^2^/g. Similar experiments but using biochar obtained from sawdust biomass were conducted by Borhan et al [25]. The activation with potassium hydroxide (the same proportion) at 500 and 600 °C was performed providing the material with the surface area of 1429.06 and 1690.32 m^2^/g [25]. The highest surface area and pore volume of the physically treated samples was obtained for a CO_2_ to carbon molar ratio of 1:1, with results of 682.1 m^2^/g: 0.35 cm^3^/g as determined for straw and 385 m^2^/g: 0.2 cm^3^/g for wood. In both experiments, a lower gas (CO_2_ and steam) to carbon molar ratio (1:0.5) provided a less extended surface area of the final material. 

According to the literature the chemical activation with KOH above 700 °C results in formation of metallic potassium which may influence the biochar structure contributing in larger specific surface area and higher volume of pores after BET analysis. Moreover, formation of the functional groups, e.g., alcohols was confirmed by FT-IR analysis [36]. The functional groups also influence the surface properties and the quality of the activated carbon. Steam or carbon dioxide used as physical activation agents, in turn, penetrate solid structure, remove volatile residues and facilitate their desorption [30]. For comparison, the physical activation of barley straw using carbon dioxide and steam was also done by Palleres et al. [37]; however, the highest surface area obtained was 800 m^2^/g for the activation of char by CO_2_ and 500 m^2^/g for steam. The gas flow was set for 2500 and 4000 cm^3^/min for 60 min for 25 g of sample, which approximates a molar ratio of 1:1 and 1:2 [37]. Ahmadpour et al. [27], in turn, performed the activation of macadamia nut shells using KOH and ZnCl_2_ with three different preparation methods at various temperatures (600, 700 and 800 °C). The results for the surface area of the products obtained after activation using KOH at 600, 700 and 800 °C were 628, 1075 and 1169 m^2^/g, respectively. The waste wood was also used in the investigation of Boguta et al. [38], who applied various activation reagents; the highest effect on surface area (an increase up to 82%) of char was obtained when 1M NaOH was used for activation. 

The morphology of the AC samples was analysed by scanning electron microscopy; the results are shown in Figure 4. The structure of a straw pellet without activation was more jagged and uneven; however, after activation, the surface became more even and with no single particle of pyrolysis products. In this regard, oxidation led to surface ordering of the material. The fibrous structure of the wood is clearly visible in the micrographs. It was observed that with increasing surface area, the number of single particles decreased and the surface became less frayed. Therefore, we confirmed that chemical activation preferably provides a well-propagated microporous structure inside the raw material particles determining an appropriate adsorption surface (Figure 4).

### 3.2. Adsorption Analysis with Rhodamine B

**Figure 5:**
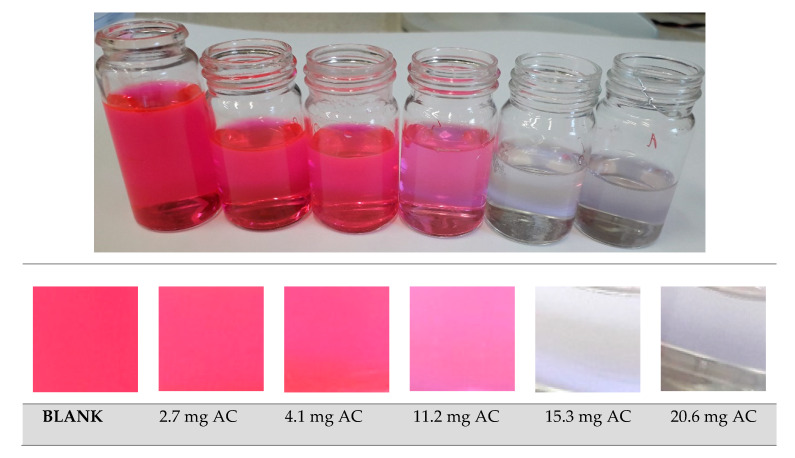
Discoloration of the RhB solution by the application of the activated carbon obtained by the pyrolysis of the straw pellet and chemical activation with KOH (the sample to KOH mass ratio of 1:2). The numbered pictures represent various dosages of activated carbon: no. 2–6 depict 2.7, 4.1, 11.2, 15.3 and 20.6 mg, respectively, used for sorption of 30 mg/dm^3^ of Rhodamine B at 25 °C.

The sorption properties of the materials were examined by using samples with the highest surface area (1349.6 m^2^/g) and total pore volume, namely the straw pellet after chemical activation with KOH. In this experiment, Rhodamine B was used as a model water contaminant to observe the efficiency of the adsorption by the activated carbon. The sorption experiment was performed using a bath technique. Various doses (2.7, 4.1, 11.2, 15.3 and 20.6 mg) of AC were mixed with the dye solution (30 mg/dm^3^). The colour of the solutions is compared in Figure 5. The first picture presents the colour of the solutions without adding activated carbon (reference experiment), whereas pictures 2–6 are of samples with the addition of 2.7, 4.1, 11.2, 15.3 and 20.6 mg of activated carbon in the vessels. In all experiments of the RhB removal, the particle size, temperature, contact time (30 min), initial concentration, pH and concentration of the other ions were kept constant. 

**Figure 6:**
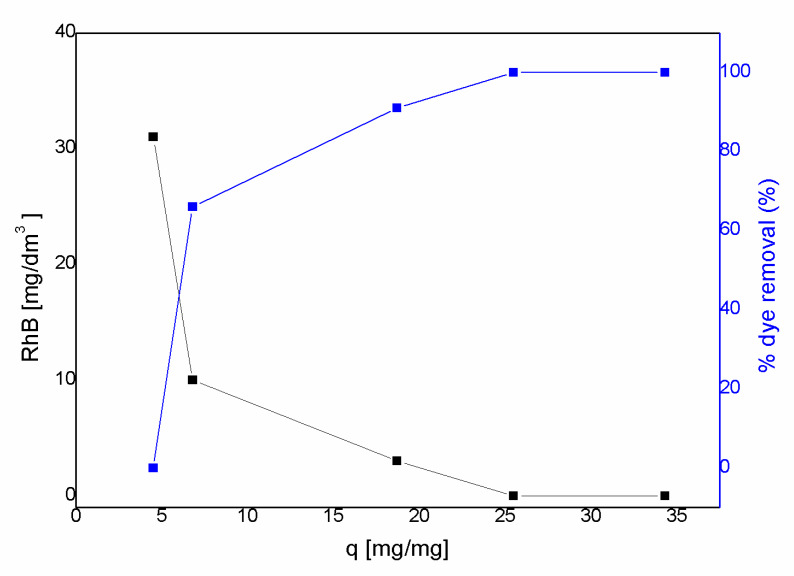
Effect of the activated carbon dosage (chemical activation 1:2, straw pellet sample) in the range 2.7–20.6 mg AC for 30 mg/dm^3^ of Rhodamine B solution, sorption at 25 °C determined by UV-VIS analysis, where: q—the activated carbon per mass of the dye adsorbed, AC/RhB. % dye removal—percentage of RhB dye adsorbed by the activated carbon (with unchanging initial concentration of dye).

The chemically activated carbon used in different quantitative proportions resulted in the discoloration of the RhB dye solutions. As expected, the effectiveness of adsorption increased with increasing amounts of the activated carbon used in the experiment until the complete discoloration of the solution. The relative proportion of the amount of RhB and the activated carbon and the results of the experiment are presented in Table 3 and Figure 6. The adsorption of the dye on the activated carbon was studied by varying the carbon concentration used for the experiments. The dependence of the amount of activated carbon per mass of dye adsorbed (q) and percentage of the dye adsorbed by the activated carbon in solution with unchanging initial concentration of dye (% dye removal) are presented in Figure 6. The percent of adsorption increased with the amount of activated carbon. Rhodamine, the model waste water contaminant, is adsorbed even by small amounts of AC, with 4.1 mg decreasing the RhB concentration by 30%. Total removal of the model solution colour can be observed when 15.3 mg AC was added. RhB was completely adsorbed on porous material and could not be detected using UV-VIS analysis. Further addition of the adsorbent did not change the colour of the solution. The experiment can be observed without using special equipment and confirms the sorption properties of the straw pellet activated material.

The adsorption of Rhodamine B from an aqueous solution of various materials was conducted by other researchers, who varied parameters: pH and temperature of solution, concentration, dosage of adsorbent and others [34,39]. It was found that the addition of hydrochloric acid may improve the efficiency of dye adsorption, and the highest adsorption of RhB was detected in the range of pH 2.0–6.0 [34]. However, the value of pH does not significantly affect the efficiency of dye adsorption. Nevertheless, pH determines formation of various ionic species in solutions and influences surface charge of the carbon [34,39]. Additionally, increasing temperature and ionic strength of the solution may also improve the dye adsorption due to enlargement of pore size, activation of the adsorbent as well as reduction of the repulsion between carbon and contaminant [34,39]. In this study authors used only one, but the most important parameter, to confirm sorption properties of AC obtained from straw biomass. 

## 4. Conclusions

The activated carbon with the highest surface area was obtained by the chemical treatment of biochar obtained from straw and wood biomass with the application of KOH solution (1349.6 and 1194.4 m^2^/g for straw and wood samples, respectively). Considering the elemental, thermogravimetric and scanning electron microscope analyses, the differences in the structure and composition of raw materials were detected. Based on these results differences in the activation were discussed. The cellular structure of straw and wood affected the activation agent penetration and surface area. The structure of the wood chips is more compact, and thus more difficult to activate, while pellets provide an example of a material that enables the flow and penetration of gaseous activation agents. A higher surface area was obtained when CO_2_ was used as an agent in comparison to steam. Moreover, it was shown that the higher the amount of the activation agent, the greater is the adsorption surface. Nevertheless, the chemical activation was more effective than the physical method. A comparison of the results determined for both biomass samples revealed that activated carbon produced from straw pellets had higher surface area regardless of the activation method.

## Figures and Tables

**Table 1:** Composition and properties of the raw waste materials used in the experiments and the chars obtained by pyrolysis.

Proximate Analysis (wt.%)					Elemental Analysis (wt.%)				Calorific Value (MJ/kg)
**Sample**	Fixed Carbon	Moisture	Ash	Volatile	C	H	N	O	
**Straw pellet**	1.8	6.7	5.9	85.6	49.0	6.9	1.7	36.6	21.3
**Char-straw Pellet**	–	–	–	–	76.2	0.7	3.4	–	
**Wood**	21.4	8.4	2.3	67.9	45.0	6.4	1.3	47.3	19.6
**Char-wood**	–	–	–	–	74.0	0.6	3.5	–	

**Table 2:** Surface area (S_BET_) and pore size (V_p_) of the activated carbon obtained from the straw pellet and waste wood.

No.	Activation Agent	Activation Agent to Carbon Molar Ratio	Straw Pellet		Wood Strips	
			S_BET_ (m^2^/g)	V_p_ (cm^3^/g)	S_BET_ (m^2^/g)	V_p_ (cm^3^/g)
1	without activation (reference sample)		3.1	0.002	27.3	0.013
2	steam	1:0.5	226.9	0.12	229.5	0.12
3	steam	1:1	452.5	0.23	248.5	0.13
4	CO_2_	1:0.5	401.2	0.21	228.8	0.12
5	CO_2_	1:1	682.1	0.35	385.0	0.20
6	KOH	1:2 *	1349.6	0.69	1194.4	0.61

S_BET_— Brunauer–Emmett–Teller (BET) surface area [m^2^/g]; V_p_—total volume of pore [cm^3^/g]; * mass ratio.

**Table 3:** The adsorption parameters.

	Before Adsorption			After Adsorption	
No.	Activated Carbon (mg)	Rhodamine B (mg)	Activated Carbon/Rhodamine B (q) (mg/mg)	Rhodamine B (mg/dm^3^)	% Dye Removal
1	0	0.6	–	30	0
2	2.7	0.6	4.5	30	0
3	4.1	0.6	6.8	10	66
4	11.2	0.6	18.7	3	91
5	15.3	0.6	25.5	n.d.	100
6	20.6	0.6	34.3	n.d.	100

n.d.—not detected.
